# What is the influence of randomisation sequence generation and allocation concealment on treatment effects of physical therapy trials? A meta-epidemiological study

**DOI:** 10.1136/bmjopen-2015-008562

**Published:** 2015-09-03

**Authors:** Susan Armijo-Olivo, Humam Saltaji, Bruno R da Costa, Jorge Fuentes, Christine Ha, Greta G Cummings

**Affiliations:** 1 *CLEAR Outcomes (Connecting Leadership, Education and Research)* Research Program, University of Alberta, Edmonton Clinic Health Academy (ECHA), Edmonton, Alberta, Canada; 2 Faculty of Rehabilitation Medicine, Department of Physical Therapy, University of Alberta, Edmonton, Alberta, Canada; 3 Faculty of Rehabilitation Medicine, Rehabilitation Research Center, University of Alberta, Edmonton, Alberta, Canada; 4 Orthodontic Graduate Clinic, School of Dentistry, University of Alberta, Edmonton, Alberta, Canada; 5 Universitat Bern, Institute of Primary Health Care, Bern, Switzerland; 6 Faculty of Rehabilitation Medicine, University of Alberta, Edmonton, Alberta, Canada; 7 Department of Physical Therapy, Catholic University of Maule, Talca, Chile; 8 Faculty of Nursing, CLEAR Outcomes (Connecting Leadership Education & Research) Research Program, University of Alberta, Edmonton Clinic Health Academy|University of Alberta, Edmonton, Alberta, Canada

**Keywords:** allocation concealment, sequence generation, physical therapy, meta-epidemiological, risk of bias

## Abstract

**Objective:**

To determine if adequacy of randomisation and allocation concealment is associated with changes in effect sizes (ES) when comparing physical therapy (PT) trials with and without these methodological characteristics.

**Design:**

Meta-epidemiological study.

**Participants:**

A random sample of randomised controlled trials (RCTs) included in meta-analyses in the PT discipline were identified.

**Intervention:**

Data extraction including assessments of random sequence generation and allocation concealment was conducted independently by two reviewers. To determine the association between sequence generation, and allocation concealment and ES, a two-level analysis was conducted using a meta-meta-analytic approach.

**Primary and secondary outcome measures:**

association between random sequence generation and allocation concealment and ES in PT trials.

**Results:**

393 trials included in 43 meta-analyses, analysing 44 622 patients contributed to this study. Adequate random sequence generation and appropriate allocation concealment were accomplished in only 39.7% and 11.5% of PT trials, respectively. Although trials with inappropriate allocation concealment tended to have an overestimate treatment effect when compared with trials with adequate concealment of allocation, the difference was non-statistically significant (ES=0.12; 95% CI −0.06 to 0.30). When pooling our results with those of Nuesch *et al*, we obtained a pooled statistically significant value (ES=0.14; 95% CI 0.02 to 0.26). There was no difference in ES in trials with appropriate or inappropriate random sequence generation (ES=0.02; 95% CI −0.12 to 0.15).

**Conclusions:**

Our results suggest that when evaluating risk of bias of primary RCTs in PT area, systematic reviewers and clinicians implementing research into practice should pay attention to these biases since they could exaggerate treatment effects. Systematic reviewers should perform sensitivity analysis including trials with low risk of bias in these domains as primary analysis and/or in combination with less restrictive analyses. Authors and editors should make sure that allocation concealment and random sequence generation are properly reported in trial reports.

**Strengths and limitations of this study**

- To the best of our knowledge, this is the first meta-epidemiological study using continuous outcomes in allied health disciplines such as physical therapy (PT).
- This large meta-epidemiological study, which was based on 43 Cochrane reviews, 393 trials and 44 622 patients makes an important contribution to the field.
- This study provides with novel evidence regarding the association between adequacy of randomisation and allocation concealment and treatment estimates in PT.
- We restricted our analysis to Cochrane systematic reviews in PT and results might not be applicable to all Cochrane reviews or other reviews conducted in other areas of research.
- For determining the association between random sequence generation and allocation concealment, we limited our analysis to trials describing a true control group, or placebo intervention, reducing our statistical power.

## Introduction

Randomisation and allocation concealment have been extensively investigated in the medical literature as key methodological characteristics of randomised controlled trials (RCTs) in medical research.^1–3^ Allocation concealment is the most commonly evaluated quality characteristic in reviews of RCTs due to its crucial role as a key marker of internal validity of RCT.^4^ Moreover, it is firmly established the conventional wisdom that adequate randomisation sequence generation is an essential part of a valid clinical trial.^5^

There has been an extensive body of empirical research looking at the influence of random sequence generation and allocation concealment on treatment effect estimates of health RCTs. Several meta-epidemiological studies investigating the association between trial characteristics and treatment effects have found an association between inadequate randomisation and/or allocation concealment and an overestimation of treatment effects.^1–3^ ^6–9^ For example, inadequate random sequence generation can overestimate treatment effects by 12% (Ratio of ORs (ROR) 0.88, 95% CI 0.79 to 0.99).^10^ Inadequate allocation concealment can overestimate treatment effects on average by 18%.^3^ ^8^ ^11^ In addition, Pildal *et al*^12^ found that 2/3 of the conclusions from meta-analyses were no longer supported if only trials with adequate allocation concealment were included and that 69% of meta-analyses lost statistical significance when trials with unclear or inadequate allocation concealment were excluded.^12^ Over-estimates or underestimates of treatment effects can lead to biased or inaccurate results and conclusions in systematic reviews and meta-analyses.^3^ ^12–15^ These factors can ultimately have repercussions on decision-making and quality of patient care since different assessments could lead to different decisions for clinical practice.

Most of the empirical evidence regarding the relationship between trial components, and specifically random sequence generation and allocation concealment, and treatment effect estimates comes from RCTs in medicine and is based mainly on dichotomous outcomes.^3^ ^8^ ^11^ No such studies using continuous outcomes have been conducted in other health areas such as the allied health professions, including physical therapy (PT). Furthermore, although it has been reported that the quality of reporting of PT trials has improved over time,^16^ a finer analysis of types of random sequence generation and allocation concealment in PT trials is lacking.

Therefore, it is necessary to determine the extent to which sequence generation and allocation concealment affect treatment effect estimates in PT trials to provide accurate results to the PT clinical community. This information is urgently needed to develop guidelines for designing, conducting and implementing PT trials as well as providing clear benchmarks to assess the quality and/or risk of bias of PT trials in systematic reviews and meta-analyses and ultimately the strength of evidence for decision-making in PT. Therefore, our research questions were: (1) are random sequence generation and allocation concealment adequately used and reported in RCTs of PT; (2) do random sequence generation and allocation concealment have an effect on estimates of treatment in PT trials; and (3) do effects sizes on PT trials differ depending on some characteristics of the meta-analyses analysed such as magnitude of the effect size, meta-analysis heterogeneity, type of outcome (subjective or objective) and whether the meta-analysis involved the musculoskeletal area.

## Method

### Design

Meta-epidemiological approach.

### Study selection

A random sample of RCTs included in meta-analyses in the PT discipline were identified by searching the Cochrane Database of Systematic Reviews from January 2005 to 25 May 2011 on PT interventions. The search strategy can be found elsewhere and can be provided on request.^17^ ^18^ Meta-analyses were included if they met the following eligibility criteria: (1) the meta-analysis included at least three RCTs comparing at least two interventions, with at least one of the interventions being part of PT scope of practice according to the World Confederation for Physical Therapy (WCPT);^19^ and (2) the main outcome or the outcome of the meta-analysis with the largest number of trials conducted in the review was continuous.

### Assessment of random sequence generation and allocation concealment domains

Assessments of random sequence generation and allocation concealment domains were performed by two independent reviewers following the Cochrane collaboration guidelines.^20^

### Random sequence generation assessment

In addition to the general evaluation of adequacy of random sequence generation, different methods of random sequence generation were determined. We grouped these methods into four categories as follows: *Category 1* included trials where random sequence generation was unclear or not reported; *category 2* included trials that had adequate randomisation (eg, use of a computer software, random number table and minimisation); *category 3* included trials using acceptable methods of randomisation, but less efficient than the previous category (eg, drawing lots, envelopes, shuffling cards, throwing a dice); *category 4* involved trials using inappropriate methods of sequence generation (eg, date of birth, day of admission, hospital record number). Categorisation into one of these four categories was conducted in duplicate. To facilitate comparison with previous meta-analyses, these four categories were combined to create two main groups: an “adequate sequence generation” group (combining categories 2 and 3) and an “inadequate or unclear sequence generation” group (combining categories 1 and 4).

### Allocation concealment assessment

We divided the methods of allocation concealment used in the trials into the following categories: *Category 1* comprised trials that used any type of central randomisation (eg, a remote telephone service or a central office); *category 2* comprised trials that used sequentially numbered, opaque and sealed envelopes**;** *category 3* comprised trials that used sealed envelopes without reporting any further details; and *category 4* comprised trials where allocation was clearly not hidden (eg, being based on an open list, odd or even days of the week, participant's birth date or the team on duty at enrolment); and *category 5* comprised trials were concealment of allocation was not reported or unclear. Categorisation into one of these five categories was conducted by two independent assessors. To facilitate comparison with previous meta-analyses, these five categories were combined to create two main groups: an “adequate concealment” group (combining categories 1 and 2) and an “inadequate or unclear concealment” group (combining categories 3, 4 and 5).

### Data extraction of treatment estimates and trial characteristics

Two independent reviewers extracted specific data (eg, type of interventions, type of outcomes (ie, objective, subjective), PT area) for all trials included in the meta-analyses as well as data on means, SDs and sample sizes. The primary outcome chosen for the analysis was the main outcome of interest reported in the review or determined from the meta-analysis that contained the largest number of trials in the review. Details on the reviewers’ panel and training process can be found elsewhere.^17^ ^18^

### Data analysis

Data on sequence generation and allocation concealment were analysed descriptively based on the categories described previously. In order to determine whether random sequence generation and allocation concealment domains affect treatment effect estimates, a two-level analysis was conducted using a meta-meta-analytic approach with a random-effects model to allow for within and between meta-analyses heterogeneity as suggested by Sterne.^21^

The first level analysis (within meta-analysis) was as follows: we derived effect sizes (ES) for each trial by dividing the between-group difference in mean values by the pooled SD.^22^ A negative ES indicates a beneficial effect of the experimental intervention. If some required data were unavailable, we used approximations as previously described.^23^ The data from each trial were obtained from the meta-analyses included in our study. We followed the classification used in the Cochrane reviews to classify the treatment arms as the experimental treatment of interest or as the control group. In the case of studies appearing in more than one review, the study was only considered once in the meta-analysis with the fewer number of overall studies. We then calculated two pooled ES for each meta-analysis: one corresponding to the pooled effect size from studies having the characteristic of interest (eg, allocation concealment) and the other for studies that did not (eg, no or unclear allocation concealment). We used standard random-effects meta-analyses to combine ES across trials and calculated the DerSimonian and Laird estimate of the variance to determine heterogeneity between trials.^1^ ^24^ Then, for each meta-analysis, we derived the difference between pooled ES estimates from trials with and without the characteristic of interest (eg, allocation concealment). A negative difference in ES indicates that trials without the characteristic of interest show a more beneficial effect for the experimental group.

The second level analysis (between meta-analyses) involved pooling the results of the previous analysis to describe the effect of each trial component across all meta-analyses. The ES were also combined at this stage using the DerSimonian and Laird random-effects models^25^ to allow for between meta-analysis heterogeneity.

Formal tests of interaction between adequate sequence generation and concealment of allocation and estimated treatment benefits were performed separately for each meta-analysis based on Z scores using the estimated difference in ES between trials with and without adequate sequence and concealment of allocation and the corresponding SE.

We additionally stratified analyses accompanied by interaction tests according to the prespecified characteristics as reported by Nuesch *et al*^1^ as follows: treatment benefit in overall meta-analysis: small (ES greater than −0.5) versus large (ES≤ to −0.5); between-trial heterogeneity in overall meta-analysis (low (τ^2^<0.06) vs high (τ^2^≥0.06)), nature of the outcome (subjective or objective) and if the intervention was classified as musculoskeletal or other PT area.

In order to evaluate the effect of random sequence generation and allocation concealment on treatment effect estimates, we limited the analyses to studies describing a true control group, or placebo intervention as well as studies in which the direction of the expected treatment effect was evident (ie, standard care vs standard care plus active intervention; and active intervention 1 plus active intervention 2 vs active intervention 1).

Finally, results of our analyses were pooled with results from the meta-epidemiological study performed by Nuesch *et al*,^1^ which also investigated the effect of concealment of allocation on treatment effects on osteoarthritis measured on a continuous scale. Stata statistical software V.12 was used to perform these analyses.

## Results

### Selection and characteristics of meta-analyses and RCTs

**Figure 1:**
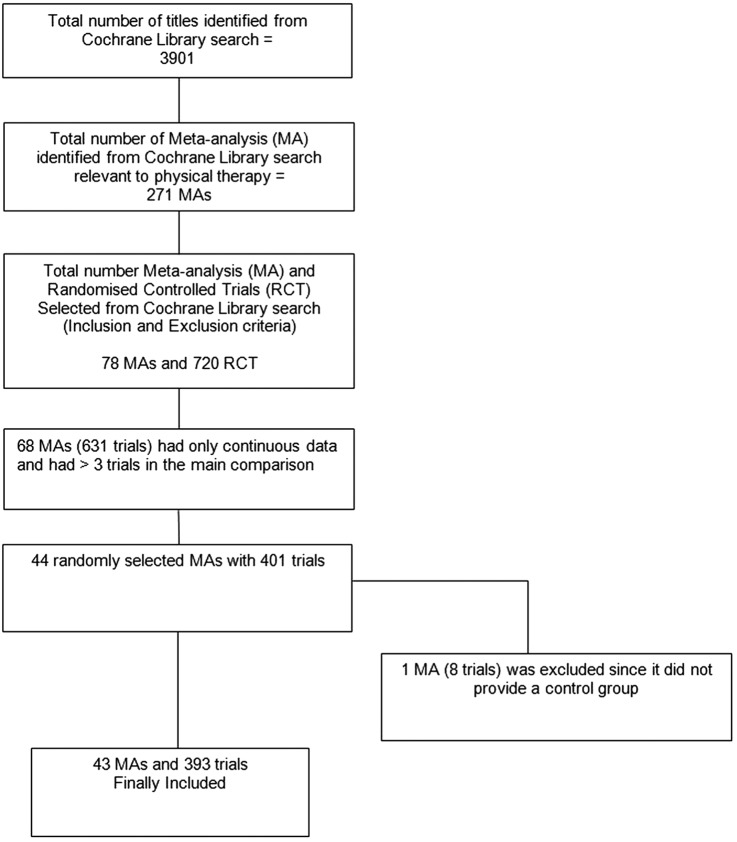
Diagram for identification of studies.

The search identified 3901 Cochrane reviews, with 271 reviews potentially relevant to PT. Of these, 68 reviews included a meta-analysis of at least three studies of PT interventions and used a continuous outcome. We randomly selected 44 meta-analyses but excluded one 26 because it used follow-up data from the same group rather than a control group for comparison (figure 1). Forty-three meta-analyses including 393 trials and analysing 44 622 patients contributed to this study. Table 1 summarises the characteristics of the 43 Cochrane reviews. Briefly, the reviews were published between 2008 and 2011 and included meta-analyses of the effectiveness of PT interventions for musculoskeletal (22 reviews),^27–35^ cardiorespiratory (9 reviews),^36–44^ neurological (6 reviews)^45–51^ and other areas of physical therapy (6 reviews).^51–56^ A median number of six trials were included in the meta-analyses (IQR 5–8). Most trials were parallel group trials (367; 93.4%), single-centre studies (298; 76%) and had active control interventions (362; 92%). The most common intervention was exercise (n=282, 71.8%). Online Supplementary table S1 (appendix S1) lists the characteristics of each of the 43 meta-analyses.

**Table 1:** Characteristics of the selected meta-analysis within physical therapy areas

	Musculoskeletal	Cardio respiratory	Neurology	Other
Characteristics				
Number of meta-analyses	22	9	6	6
Median year of publication	2009	2010	2009	2009
Total number of trials included	194	78	52	69
Total number of patients included	19 861	9015	2138	13 608
Main intervention				
Exercise	13	7	3	5
Physical agents	1	0	1	0
Acupuncture	2	0	0	0
Manual therapy	1	0	0	0
Other	1	2	2	1
Outcomes				
Clinician-assessed outcome	8	4	6	3
Self-reported outcome	11	4	0	2
Administrative data or automated outcome	3	1	0	1

### Sequence generation descriptive

From the 393 trials included in the 43 meta-analyses, 156 trials (39.7%) had appropriate sequence generation and 237 (60.3%) had inappropriate sequence generation (229 were classified as ‘unclear’ and 8 as ‘high’ risk of bias) according to the Cochrane Collaboration guidelines.

When analysing the sequence generation categories, we found that 229 trials (58.27%) did not clearly report the mechanism of how the sequence was generated. One hundred and thirty-two trials (33.6%) generated the sequence through computer software, a table of random numbers or by minimisation method. For a more specific sequence generation methods description, see table 2.

**Table 2:** Methods of sequence generation in physical therapy trials

Sequence generation methods	Number of trials (%)
Unclear/not reported	232 (59.03)
Computer software	65 (16.54)
Random number table	60 (13.49)
Drawing of lots	12 (3.05)
Shuffling cards or envelopes	9 (2.29)
Minimisation	6 (1.52)
Other non-random methods	4 (4.33)
Coin tossing	1 (0.25)
Date of birth	1 (0.25)
Day of admission	1 (0.25)
Hospital/institution records number	1 (0.25)
Throwing a dice	1 (0.25)
Total	393 (100)

In addition, simple randomisation was reported in 34 trials, block randomisation in 54 trials, stratification in 62 trials and unclear or not reported in 218 trials. The rest of the trials reported other methods of random sequence generation.

### Allocation concealment descriptive

From the 43 meta-analysis and 393 trials, 45 trials (11.5%) had appropriate allocation concealment and 348 (88.6%) had inappropriate concealment of allocation according to the Cochrane Collaboration guidelines. Results of analysis of the allocation concealment within each category were as follows: 282 trials (71.8%) had unclear method of allocation concealment, 21 (5.34%) trials used central allocation, 24 (6.11%) trials used envelopes with all 3 safe wards (opaque, sealed and sequentially numbered), 15 (3.82%) trials used envelopes without safeguards and 51 (16.8%) trials used any other non-concealed methods. Further details on allocation concealment methods are displayed in table 3.

**Table 3:** Methods of allocation concealment in physical therapy trials

Allocation concealment methods	Number of trials (%)
Unclear/not reported	282 (71.76)
Central concealment	21 (5.34)
Sequentially numbered, opaque and sealed envelopes	24 (6.11)
Unsafe envelopes	15 (3.82)
Non-safe methods of allocation	51 (12.98)
Total	393 (100)

### Sequence generation and allocation concealment descriptive

Only 8.9% of the trials (n=35) had both appropriate sequence generation and appropriate allocation concealment. A great percentage (51%; n=199) of the trials did not have either appropriate sequence generation or appropriate concealment of allocation.

### Sequence generation and treatment effects in PT trials

**Figure 2:**
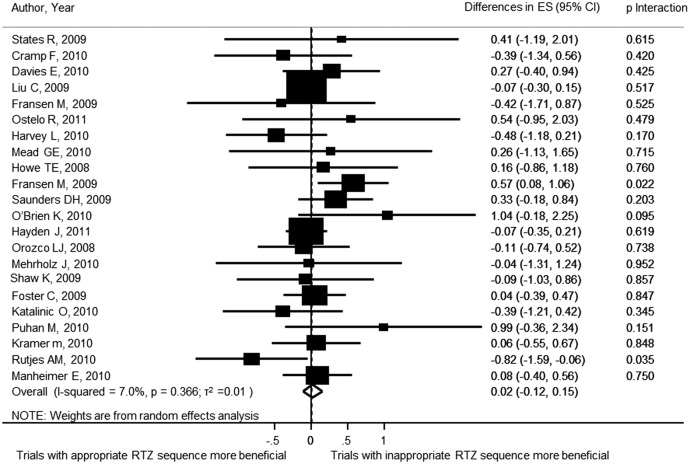
Forest plot of the differences in effect sizes between trials with and without adequate sequence generation.

**Figure 3:**
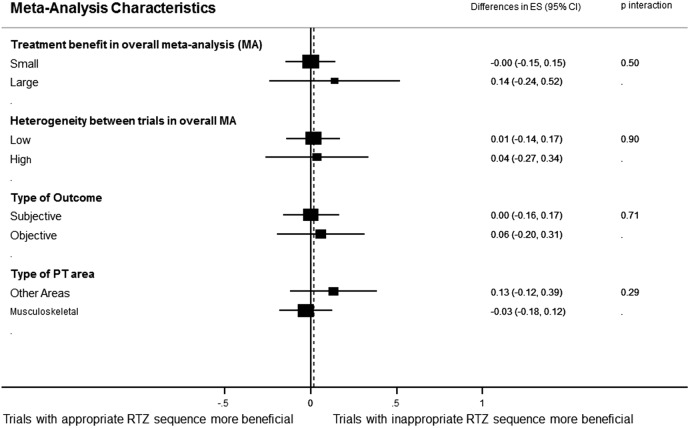
Forest plot of the differences in effect sizes between trials with and without sequence generation stratified by meta-analyses characteristics (effect size magnitude, heterogeneity, type outcome and physical therapy area).

For the purpose of analysing the effect of sequence generation on treatment effects, 22 meta-analyses including 257 trials and analysing 30 287 patients contributed to this analysis. Figure 2 shows the forest plot of the differences in ES between trials with adequate and inadequate random sequence generation. There was no statistically significant difference between the ES of trials with adequate or inadequate random sequence generation (ES=0.02; 95% CI −0.12 to 0.15). The results of the stratified analyses are displayed in figure 3. None of the meta-analyses characteristics had a statistically significant interaction.

When analysing trials belonging only to *category 2* which included trials that had adequate sequence generation (eg, use of a computer software, random number table and minimisation) versus unclear or not reported sequence generation, no statistically significant difference between the ES of these trials was found (ES=0.10; 95% CI −0.04 to 0.23). However, trials with unclear or not reported sequence generation tended to overestimate the treatment effect when compared with trials with adequate sequence generation from category 2. It was not possible to conduct the subgroup analysis between trials of category 2 versus trials from *category 4* (using inappropriate methods to perform sequence generation such as date of birth, day of admission, hospital record number) since there were not enough trials for such analysis.

### Allocation concealment and treatment effects in PT trials

**Figure 4:**
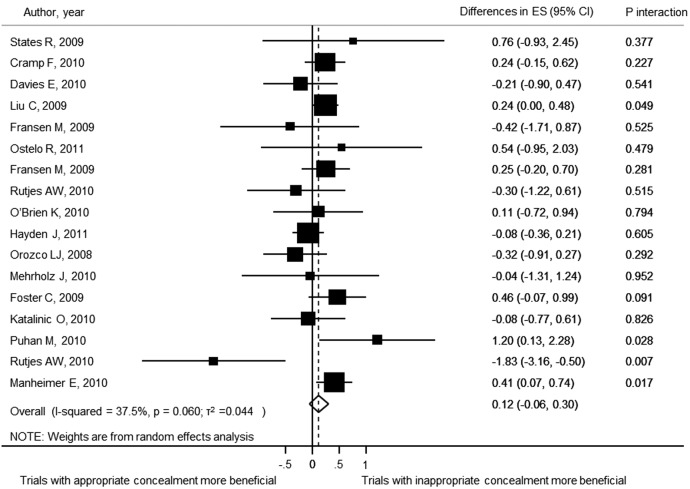
Forest plot of the differences in effect sizes between trials with and without adequate concealment of allocation.

**Figure 5:**
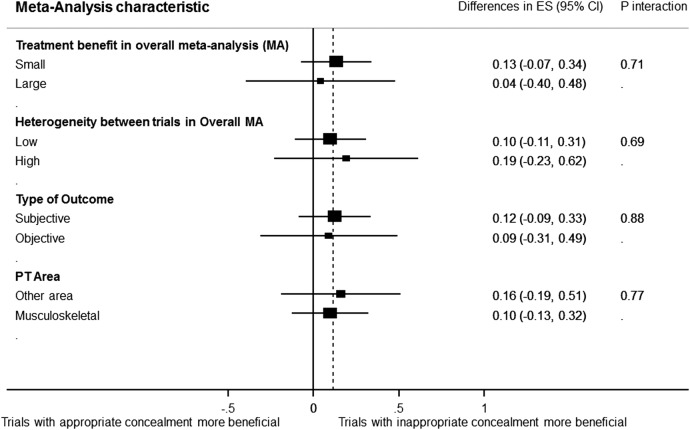
Forest plot of the differences in effect sizes between trials with and without adequate concealment of allocation stratified by meta-analyses characteristics (effect size magnitude, heterogeneity, type outcome and physical therapy area).

For the purpose of analysing the effect of allocation concealment on treatment effects, 17 meta-analyses including 198 trials and analysing 27 011 patients contributed to this analysis. Figure 4 shows the forest plot of the differences in ES between trial with adequate and inadequate allocation concealment. Although trials with inappropriate allocation concealment tended to have an overestimate treatment effect when compared with trials with adequate concealment of allocation, the difference was non-statistically significant (ES=0.12; 95% CI −0.06 to 0.30). The results of the stratified analyses are displayed in figure 5. None of the meta-analyses characteristics had a statistically significant interaction. When focusing on trials with appropriate allocation concealment (category 1 and 2) versus trials with clearly inappropriate methods of concealment (category 4), the difference was not statistically significant (ES=0.26; 95% CI −0.04 to 0.55). However, trials with clearly inappropriate methods of concealment (category 4) tended to overestimate the treatment effects. The same was the case when comparing the trials with appropriate allocation concealment (category 1 and 2) versus trials with unclear or unreported concealment of allocation (ES=0.20; 95% CI 0.05 to 0.34). This time, the difference between these trials was statistically significant.

### Pooling results with a previous meta-epidemiological study

**Figure 6:**
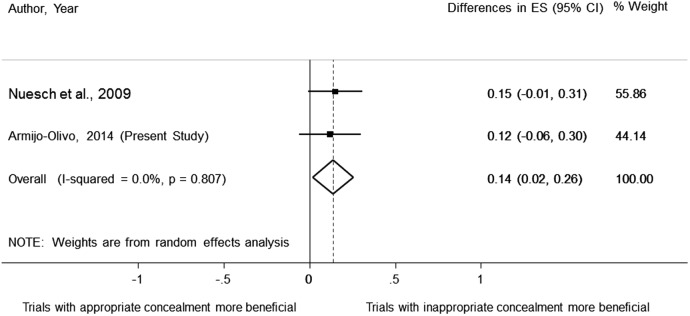
Pooled data of the effect of concealment of allocation on treatment effect estimates using continuous outcomes.

When pooling our results with those of Nuesch *et al*,^1^ who performed a meta-epidemiological study with continuous outcomes as well, we obtained a pooled statistically significant value (ES=0.14; 95% CI 0.02 to 0.26), meaning that trials with inappropriate concealment of allocation had more beneficial effect than trials with appropriate concealment of allocation (figure 6).

## Discussion

Our findings showed that adequate sequence generation and appropriate allocation concealment were only accomplished in a low percentage of PT trials (39.7% and 11.5%, respectively). In addition only 8.9% of the trials (n=35) had both appropriate sequence generation and appropriate allocation concealment despite the fact that both of the aforementioned risk of bias domains can be always appropriately performed when conducting an RCT in any field.^57^ In addition, trials with inappropriate concealment of allocation had an overestimation of treatment effects when compared with trials with adequate concealment of allocation. However, no difference in treatment effects in trials with appropriate or inappropriate sequence generation was found. These results confirm previous results obtained by several meta-epidemiological studies investigating the influence of sequence generation and allocation concealment on several areas of medicine using dichotomous outcomes.^7^ ^8^ ^11^ ^12^ ^58^ In addition, the pooled estimate obtained from our study and that of Nuesch *et al*,^1^ indicated that trials with inappropriate allocation concealment had a more beneficial effect than those with appropriate allocation concealment.

These results have important implications for the research community in general as well as for the discipline of PT. To our knowledge; this study is the first of its kind conducted in PT that examined continuous outcomes. Thus, it provides novel evidence in a very specific area of health. Most of the previous studies were looking at medical areas, such as pregnancy and childbirth, circulatory conditions, infectious disease, surgery and mental health trials. All of these medical areas certainly differ from PT with respect to: type of intervention (being mostly drugs), type of outcomes used and specific area of study.

First, it is not surprising that a large number of trials did not clearly report either randomisation sequence or allocation concealment or both. Our findings showed that approximately 62% and 70% of studies were classified as ‘unclear/not reported’ in their reporting of sequence generation and allocation concealment, respectively. This has also been observed in a meta-epidemiological study investigating meta-analyses before 2005.^2^ The study found a 64% and 69% of ‘unclear’ reporting of sequence generation and allocation concealment, respectively.^2^ Thus, despite the efforts made to improve reporting of RCTs such as introducing the CONSORT Statement, there is still inappropriate reporting and implementation of these important characteristics in RCTs. This is in agreement with results reported by Moseley *et al*,^16^ who found little or no effect of the CONSORT Statement on the quality of reports of physiotherapy trials. Quality of reporting has always been an issue when trying to understand the association between trial characteristics and treatments effects since most of authors do not properly report their methods. Although inappropriate reporting does not always reflect on weak trial conduct,^59^ some studies such as Pildal *et al*,^12^ found that most trials with unclear allocation concealment had also unclear allocation concealment in their protocol. Therefore, quality of reporting is related to assessment of risk of bias, but the direction of this relationship is still unclear. Future studies should assess how, how much and in which direction trial reporting affects treatment effects.

The magnitude and direction of the effect found in our study is consistent and almost identical with a previously published study performed in osteoarthritic trials investigating allocation concealment. In our study, the average bias associated with lack of allocation concealment corresponds to ¼ or ½ of a typical treatment effect found in PT interventions.^60–63^ Furthermore, the overestimation of a treatment effect was even higher when only focusing the analysis to trials with clearly inappropriate methods of allocation concealment. As stated by others,^2^ ^8^ if allocation concealment is not accomplished, it is more likely that the results of the trial are inaccurate because, intentionally or unintentionally, investigators could have interfered with assigning participants to different groups, favouring the effect of the intervention. In addition, allocation concealment could be a proxy measure for other aspects of trial design besides selection bias.^2^ ^8^

Random sequence generation was found not to be significantly associated with distortion of treatment effects in this study. Thus, it appears that random sequence generation does not have such a strong influence on treatment effects in comparison with concealment of allocation based on the results of the present study. Other meta-epidemiological studies have found similar results. 8 11 64 One of these studies, performed by Balk *et al*,^64^ has been questioned because of the high heterogeneity between trials included in meta-analysis. This could introduce a higher noise to be able to detect any effect of the trials characteristics. The other studies reported the same results argue that sequence generation is still an important characteristic that ensures rigour in the trial by allowing a trial to be concealed.^8^ ^11^ In addition, other authors have suggested that the relationship between certain methodological characteristics and treatment effects varies according to each health area^64^ ^65^ and more work should be carried out in other health areas to confirm these results.^64^ ^65^

Our stratified analysis for sequence generation and allocation concealment showed that none of the meta-analysis characteristics presented with significant interaction. This is somehow contrary to other meta-epidemiological studies. For example, Wood *et al*,^3^ and Savovic *et al*,^2^ found that there was an overestimation of treatment effects that ranged between 22% and 31% for subjective outcomes. We followed exactly the same definition for determining outcomes as ‘objective and subjective’ used by Wood *et al*,^3^ to facilitate comparisons and avoid misclassifications. Thus, it seems that for PT area, there is no evidence of bias regarding random sequence generation or allocation concealment when subjective or objective outcome are used. This could be attributed to the fact that both domains can be performed in a trial regardless of the outcome analysed. Thus, whether the outcome is objective or subjective does not affect whether it is easier or not to predict patient's prognosis at the time of recruitment.

### Study limitations

We analysed the influence of random sequence generation and allocation concealment on treatment effect estimates. It could be possible that other biases interact with these biases and have an influence in the treatment effects. This should include a multivariate analysis where several biases should be taken into consideration. Future research should look into this. However, such analysis would require meta-analyses with a large number of included studies, which are extremely rare; this makes such an analysis hard to be performed.

We limited our analysis to trials describing a true control group (ie, group receiving no treatment, or a waiting list), or placebo intervention as well as those studies in which the direction of expected treatment effect was evident. In this way, we could anticipate the direction of the bias in analyses. Combining effects of all trials without clear direction of what would be the effect (ie, in case of 2 similar active interventions) would have increased noise in the analyses and heterogeneity limiting our ability to find any significant effect of trials characteristics.^66^ However, limiting our analyses to these trials, we also reduced the power of our study.

## Conclusions

Trials with inappropriate concealment of allocation had an overestimation of treatment effects when compared with trials with adequate concealment of allocation. Our results suggest that when evaluating risk of bias of primary RCTs in PT area, systematic reviewers and clinicians implementing trial findings into their clinical practice should pay attention to these characteristics, especially allocation concealment since it can be associated with an exaggeration of a treatment effect. Systematic reviewers should perform sensitivity analysis including trials with low risk of bias in these domains as primary analysis and/or in combination with less restrictive analyses. In addition, appropriate methods of sequence generation as well as allocation concealment should be implemented. Authors and editors should make sure that allocation concealment and random sequence generation are properly reported in trial reports.
